# Acute Effects of Oral Microbial Protease Co-ingestion with Whey Protein on Postprandial Plasma Amino Acid Concentrations, Appetite, and Satiety in Healthy Adults: A Randomized, Double-Blind, Placebo-Controlled, Crossover Clinical Trial

**DOI:** 10.1016/j.tjnut.2025.07.006

**Published:** 2025-07-14

**Authors:** Yijia Huang, Zachary W Bell, Alyasamin Alhamwi, Benjamin Sauvageau, Divine Malenda, Silar Gardy, Thalia Krauth-Ibarz, Sarkis J Hannaian, José A Correa, Ari Gritsas, Sean M Garvey, Kelly M Tinker, Sidney Abou Sawan, José A Morais, Tyler A Churchward-Venne

**Affiliations:** 1 Department of Kinesiology and Physical Education, McGill University, Montréal, Quebec, Canada; 2 Department of Clinical Health and Applied Sciences, University of Houston-Clear Lake, Houston, TX, United States; 3 Research Institute of the McGill University Health Centre, Montréal, Quebec, Canada; 4 Department of Mathematics and Statistics, McGill University, Montréal, Quebec, Canada; 5 Department of Research and Development, BIO-CAT, Inc, Troy, VA, United States; 6 Iovate Health Sciences International, Oakville, Ontario, Canada; 7 Division of Geriatric Medicine, McGill University, Montréal, Quebec, Canada

**Keywords:** dietary protein, digestive enzymes, microbial protease, whey protein, digestion, amino acids, appetite, satiety

## Abstract

**Background:**

Proteases are enzymes that breakdown proteins into peptides and amino acids. When co-ingested with dietary protein, proteases may enhance digestion, increase postprandial plasma amino acid concentration, and affect gut hormones, appetite, and/or satiety.

**Objectives:**

The aim of this study was to assess the effects of a mixture of 3 microbial protease preparations (P3) on postprandial plasma amino acid concentration when co-ingested with whey protein concentrate (WPC) in healthy young adults.

**Methods:**

P3 was first tested in vitro for proteolytic effects in a static simulation of orogastric digestion. In a subsequent randomized, double-blinded, placebo-controlled crossover study, 12 males and 12 females [body mass index (BMI)—mean: 23.6 (SD: 2.9); age—mean: 25 y (SD: 3 y)] consumed WPC (25 g protein) containing P3 or placebo (maltodextrin). Plasma amino acid, glucose, insulin, and appetite regulatory hormone concentrations were assessed at baseline and throughout a 240-min postprandial period. Perceived appetite sensations were assessed by visual analog scale questionnaires. An ad libitum meal was administered following each treatment to determine energy intake.

**Results:**

P3 demonstrated proteolytic activity at 50,000 hemoglobin units on tyrosine basis per 31.9 g serving of WPC in vitro. Adjusted geometric mean postprandial plasma 60-min incremental area under the curve was 14% greater for essential amino acids (treatment: *P* = 0.025) and 15% greater for branched-chain amino acids (treatment: *P* = 0.021) with P3 than placebo, with no differences for total amino acids or leucine (all *P* > 0.05). Adjusted geometric mean postprandial plasma ghrelin was 12% lower (treatment: *P* < 0.001), whereas adjusted mean visual analog scale–derived fullness (treatment: *P* = 0.025) and satiation (interaction: *t* = 30–150 min; all *P* < 0.05) were greater with P3 than placebo. Adjusted mean postprandial plasma glucose, insulin, and ad libitum meal energy intake were not different between treatments (all *P* > 0.05).

**Conclusions:**

Co-ingestion of WPC with P3 can enhance early postprandial plasma aminoacidemia and alter select indices of appetite and satiety in young adults.

This trial was prospectively registered at clinicaltrials.gov as NCT05957185.

## Introduction

Dietary protein is critical to support optimal growth, development, physiological function, and human health [[1], [2], [3]]. Following ingestion, dietary protein is digested by endogenous proteases, enabling amino acid absorption from the gastrointestinal (GI) tract. Although a substantial proportion of the absorbed amino acids are captured and metabolized by splanchnic tissues, the majority enter systemic circulation where they become available for uptake by peripheral tissues (e.g., skeletal muscle). Amino acids taken up by tissues can serve as building blocks for the synthesis of new proteins throughout the body. For example, the ingestion of dietary protein stimulates muscle protein synthesis rates and protein accretion, thereby replacing protein and amino acids lost in the postabsorptive state [4]. In addition to supporting protein synthesis, dietary protein plays an important role in satiety regulation and food consumption control by elevating plasma amino acid concentrations [5]. The presence of amino acids in the GI system triggers the release of the anorexigenic hormones glucagon-like peptide (GLP)-1 and peptide (P)YY, regulating satiation and promoting feelings of fullness and satisfaction [[5], [6], [7]]. There are, however, scenarios (e.g., aging or disease) in which effective digestion and absorption of dietary protein may be impaired [2,[8], [9], [10], [11]], thereby reducing both the postprandial increase in circulating amino acid availability, and their assimilation by peripheral tissues such as skeletal muscle. One strategy that is often considered is to simply increase the quantity of protein that is consumed with each meal, thus heightening the quantity of amino acids for absorption. However, this approach may present practical challenges for older adults, as well as individuals with GI-related disorders, who may be unable to consume larger meals or who may reduce food intake at the next meal as a result of protein-mediated satiety [[12], [13], [14]]. Another dietary approach that may enhance protein digestion and postprandial plasma aminoacidemia is co-ingestion of dietary protein with microbial proteases.

In vitro studies have shown microbial proteases function well when exposed to acidic conditions typical of the human stomach, promoting accelerated release of free amino acids and peptides from ingested dietary protein [[15], [16], [17], [18], [19]]. In vivo human research using microbial proteases as a nutritional strategy to enhance the postprandial increase in blood amino acid concentration have either shown a benefit [[20], [21], [22]] or no difference [23,24] when compared with a placebo. For example, Oben et al. [20] reported an increase in total serum amino acid concentration following co-ingestion of 50 g whey protein concentrate (WPC) with fungal proteases in 41 healthy young males. Similarly, Paulussen et al. [22] found an increase in postprandial plasma amino acid incremental area under the curve (iAUC) over the initial 120-min postprandial period following of 25 g pea protein isolate with a mixture of 3 microbial protease preparations (P3) in 24 healthy young adults. Alternatively, Townsend et al. [23] reported no difference in postprandial plasma amino acid concentration after resistance exercise following co-ingestion of 26 g whey protein with a mixture of bacterial protease and bromelain compared with whey protein alone in 10 resistance-trained males. Given these inconsistent findings, and the lack of studies to date examining the effect of P3 co-ingestion with whey protein, further studies are warranted to determine the effects of protease co-ingestion with whey protein on postprandial plasma aminoacidemia in humans.

The first aim of this study was to confirm the appropriate dosage of P3 for WPC digestion in the INFOGEST in vitro static simulation of orogastric digestion. Next, we performed a human clinical research study to assess the effect of co-ingesting WPC (containing 25 g protein) with P3 on postprandial plasma total amino acid (TAA), essential amino acid (EAA), branched-chain amino acid (BCAA), and leucine concentrations in healthy adult participants compared with that of WPC co-ingested with a maltodextrin placebo. A secondary aim was to assess the effect of co-ingesting WPC with P3 on postprandial plasma glucose, insulin, and gut-derived appetite regulatory hormone concentrations (i.e., GLP-1, PYY, and ghrelin), subjective appetite sensations, ad libitum meal energy intake, and GI tolerability in healthy adult participants compared with that of WPC co-ingested with a placebo. Given Paulussen et al. [22] recently reported that co-ingestion of pea protein with P3 increased plasma amino acid iAUC over the initial 120-min, but not the total 300-min postprandial period compared with co-ingestion of pea protein with a placebo, we hypothesized that plasma amino acid iAUC would be greater after co-ingestion of WPC with P3 than that after WPC with a placebo over the initial 120-min postprandial period. We further hypothesized co-ingestion of WPC with P3 would increase satiety and augment postprandial plasma gut hormone concentrations compared with WPC with a placebo.

## Methods

### Investigational products

OPTIZIOME P3 HYDROLYZER (Lot No. OPTIP3HOT-ZH23; hereafter referred to as P3; BIO-CAT) is a powder mixture of 3 distinct nongenetically engineered, wild-type microbial protease preparations obtained from *Aspergillus oryzae* and *Aspergillus melleus*. The protease preparations contain diluents (i.e., tapioca maltodextrin or potato dextrin) and are standardized on fungal protease activity, expressed as hemoglobin units on the tyrosine basis (HUT). One HUT unit of protease activity is the amount of enzyme that, in 1 min under the specified conditions, produces a hydrolysate from bovine hemoglobin at pH 4.7 whose absorbance at 275 nm is the same as that of a solution containing 1.10 μg/mL tyrosine in 0.006 N hydrochloric acid [25]. P3 was investigated in this study at a dose of 50,000 HUT per serving of 31.9 g WPC consisting of 25 g protein based on the sum total of measured amino acids. The P3 dose was selected based on *1*) previous clinical trial results showing that P3 at a dose of 31,875 HUT per 25 g pea protein isolate enhanced postprandial plasma aminoacidemia [22] and *2*) in vitro dose-ranging digestion simulations with whey protein that suggested a higher dose of P3 was needed to approximate the release of free amino acids from whey protein than pea protein [26]. Porcine pepsin (PP) from gastric mucosa (Product No. P6887; MilliporeSigma) was used to model endogenous human gastric pepsin activity. WPC (Product Name: Instant Whey Protein Concentrate 80, Lot No. ML22327A4; Milk Specialties Global) was used as the substrate. Results of the proximate and amino acid analyses of the WPC were provided by the product manufacturer and are shown in Table 1.

**TABLE 1:** Nutrient content of the whey protein concentrate ingested by the study participants.

Nutrient	100 g	31.9 g
Energy (kcal)	388	123.77
Moisture (g)	5.5	1.75
Crude fat (g)	7	2.23
Carbohydrates (g)	4	1.28
Crude protein (g)	83	26
Total amino acids (g)	77	25
Amino acids (g)		
Alanine	3.91	1.25
Arginine	1.98	0.63
Aspartic acid/asparagine	8.48	2.71
Cyst(e)ine	1.55	0.49
Glutamine/glutamic acid	13.12	4.19
Glycine	1.44	0.46
Histidine	1.41	0.45
Isoleucine	4.68	1.49
Leucine	8.15	2.60
Lysine	7.06	2.25
Methionine	1.48	0.47
Phenylalanine	2.46	0.78
Proline	4.32	1.38
Serine	3.86	1.23
Threonine	5.30	1.69
Tryptophan	1.39	0.44
Tyrosine	2.22	0.71
Valine	4.49	1.43

Results of the proximate and amino acid analyses of the whey protein concentrate were provided by the product manufacturer.

### In vitro digestion simulations

The static INFOGEST simulation of orogastric digestion has previously been described [27]. Briefly, the protocol models human oral and gastric phases of digestion, with a 2-min oral phase with starting pH 7 and a 120-min gastric phase with PP (2000 U/mL) and starting pH 3. To prepare the oral phase, a partial 1/30th dose of P3 was prepared in 1 mL deionized water and combined with a 9-mL solution containing 1 g WPC (equivalent to ∼1/30th serving size) in a 100-mL glass beaker. A negative control (NC), excluding both PP and P3, helped understand any effects of acid hydrolysis alone. P3 was also tested without PP to evaluate any potential interfering effect of PP on P3 function. Beakers were placed in a 20-L PolyScience water bath (Model No. WBE20A11B; Preston Industries) on a submersible stir plate (Cimerec iTelesystem 6 Multipoint Stirrers; Thermo Fisher Scientific) at 37 °C. Then, 10 mL of simulated salivary fluid was added to the WPC solution (1:1 v/w ratio) with magnetic stirring for 2 min at 200 rpm. To initiate the 120-min gastric phase, 20 mL of simulated gastric fluid with PP was added to the oral digesta (1:1 v/v ratio) and adjusted to pH 3.00 ± 0.02 using 1 N HCl. At the end of the gastric phase, digesta samples were transferred to 50-mL conical tubes and placed in a 90°C water bath for 10 min to inactivate enzymes. After cooling to room temperature, digestas were refrigerated at 4 °C before analytical testing. Experiments were performed in triplicate.

Protein hydrolysis after digestion simulations was indirectly determined by the measurements of free amino acids and free amino nitrogen (FAN) in gastric digestas. The 20 dietary proteinogenic amino acids were measured using an Agilent 1200 Series HPLC (Agilent Technologies) with a ZORBAX Eclipse Plus C18 column (4.6 × 150 mm; Part No. 959993-902; Agilent Technologies). Standards, sample preparation, elution conditions, and derivatization have been described previously [28]. FAN was measured using the nitrogen by *o*-phthaldaldehyde method [19]. Results are reported in milligrams of amino acids or milligrams of amino nitrogen per gram WPC.

### Human clinical trial

#### Study design and ethical approval

A randomized, double-blind, placebo-controlled, crossover study of healthy, recreationally active, young adults was conducted from August 2023 to January 2024 at a single research site (McGill University, Montréal, Quebec, Canada) to test the acute effects of WPC co-ingestion with P3 compared with those of WPC co-ingestion with a placebo on postprandial plasma amino acid concentrations. The clinical trial consisted of an online screening visit, a secondary in-person screening visit, and 2 in-person treatment visits. All visits conformed to standards for the use of human participants in research as outlined in the tenets of the Helsinki Declaration, and all study procedures were reviewed and approved by the McGill University Faculty of Medicine and Health Sciences institutional review board (IRB No. A05-M24-23A). The study was prospectively registered at clinicaltrials.gov as NCT05957185.

#### Sample size calculation

Target enrollment of 24 participants was based on a power analysis for the primary outcome measure [iAUC for postprandial plasma TAA concentration (μmol·L^−1^·120 min)]. Sample size was determined based on an a priori power calculation performed using G∗Power (version 3.1.9.7) [29] for estimating likely differences between total iAUC for plasma TAA concentrations [22]. Determination of iAUC effect sizes between protein and protein with P3 produced a medium effect (*d* = 0.54). To detect a medium effect size at 80% power, the analysis indicated that a sample size of 23 participants would be required for a 1-tailed *t* test between means with α at 0.05. Accounting for 1 participant to withdraw from study, 24 participants were randomly assigned into the study.

#### Participants

Twenty-four recreationally healthy adults (12 females, 12 males) were recruited from the local Montréal area through social media, word of mouth, and flyers posted at McGill University. All participants were informed about the experimental procedures, the purpose of the study, and potential risks before providing informed written consent. Participants were excluded if they had obesity, diabetes, cancer, active infections, eating disorders, irregular menstrual cycles, lactose intolerance, or any other medical conditions. Individuals were also excluded if they followed a diet that restricted dairy consumption, smoked, or were taking medications that could interfere with their ability to complete the study. All participants were deemed healthy and physically active (a minimum of 150 active min per week) based on their responses to a routine medical questionnaire and the 3-Factor Eating Questionnaire Revised 18-item version, during screening. The questionnaire was applied to assess aspects of eating behavior, namely cognitive restraint, emotional eating, and uncontrolled eating. Upon confirmation of eligibility, participants were randomly assigned and counterbalanced for enrollment. Female participants completed their treatments between day 5 and day 18 of the menstrual cycle. All participants received financial remuneration for their time following study completion.

#### Preliminary testing

After the online screening, participants underwent an in-person screening visit for measurement of height, weight, blood pressure, and body composition by dual-energy X-ray absorptiometry (GE Healthcare). Questions regarding physical activity and exercise preferences were used to determine daily habitual activity status.

#### Diet and physical activity

Study participants were asked to avoid strenuous physical activity, refrain from alcohol consumption, and wear a GT9X ActiGraph accelerometer (ActiGraph) on the wrist for 2 d immediately prior to each experimental treatment visit. Each participant was instructed to maintain their regular diet throughout the study and record their food intake in a dietary log for 2 d prior to each treatment visit. At completion of the first treatment visit, a copy of the dietary log was returned to participants. The participants were instructed to repeat their previously logged dietary intake in the 2 d immediately preceding the second treatment visit. The food logs were checked and reviewed to ensure compliance using commercially available software (Food Processor version v11.9; ESHA Research). The night before the treatment visits, participants were instructed to consume a standardized dinner at 20:00 h, after which they were instructed to remained fasted until testing the following morning. The standardized dinner (Michelina’s Beef and Macaroni; Bellisio Foods) provided 2134 kJ of energy and consisted of 52% energy from carbohydrate, 31% energy from fat, and 17% energy from protein. A reminder email was sent 1–2 d before the treatment visits.

#### Experimental protocol

**FIGURE 1:**
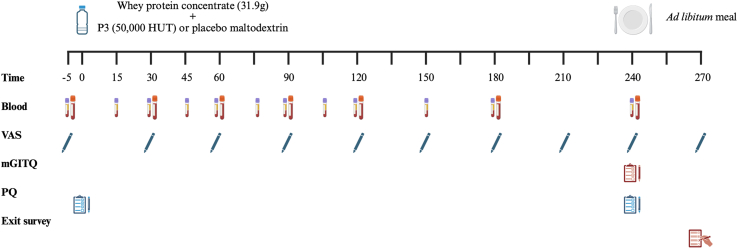
Schematic representation of the randomized, double-blinded, placebo-controlled, crossover aminoacidemia clinical trial design. HUT, hemoglobin unit tyrosine basis (fungal protease activity unit); mGITQ, modified Gastrointestinal Tolerance Questionnaire; P3, mixture of 3 microbial protease preparations; PQ, palatability questionnaire; VAS, visual analog scale. Figure created using BioRender.

A schematic representation of the experimental design is shown in Figure 1. Participants arrived at the laboratory in the morning at 08:00 in the overnight postabsorptive state for the treatment visits. During the treatment visits, participants remained in bed (except to use the washroom and consume the ad libitum meal) and were instructed to not read, talk about, or watch any food-related content. Participants were allowed to work on electronic devices or read a book during the 240-min postprandial period. The participants rested in a 45° sitting position, and a polytetrafluoroethylene catheter was inserted into a vein near the antecubital fossa and the arm was placed under a heated (60 °C) blanket for arterialized venous blood sampling. The participants then rested for 10 min before completing a paper-based visual analog scale (VAS) questionnaire to assess subjective appetite sensations (*t* = −5 min). Immediately following completion of the VAS questionnaire, a baseline arterialized venous blood sample was collected (12 mL; *t* = −5 min). Participants were then provided a freshly prepared protein beverage (31.9 g WPC powder in 250 mL water) containing the contents of 1 P3 or placebo capsule and instructed to consume the beverage within 5 min (*t* = 0 min). Further, 50 mL water was used to rinse the shaker bottle to ensure no protein powder remained. The amount of additional water ingested by the participants was controlled in both treatments. Participants then completed a palatability questionnaire (PQ) regarding the test beverage. Arterialized venous blood samples were then drawn at *t* = 15, 30, 45, 60, 75, 90, 105, 120, 150, 180, and 240 min in the postprandial period following test beverage intake. Arterialized venous blood samples were collected using prechilled blood collection tubes (BD P800 blood collection tube; 8 mL) containing a proprietary cocktail of protease inhibitors specifically optimized for stabilization of metabolic markers, and BD Vacutainer blood collection tubes (4 mL) coated with EDTA (Becton, Dickinson and Company). The VAS questionnaires were then completed immediately after blood draws at *t* = 30, 60, 90, 120, 150, 180, 210, and 240 min after beverage intake. The modified Gastrointestinal Tolerance Questionnaire (mGITQ) was completed at 240 min after the final blood draw and before the ad libitum test meal. The ad libitum meal was provided at 240 min after beverage intake. Food consumption was recorded and used to calculate energy intake. A second PQ, regarding the ad libitum meal, was administered immediately following meal consumption. At the end of the second treatment visit, participants completed an exit survey where they were asked which treatment (i.e., P3 or placebo) they thought they received during each treatment to evaluate the success of the blinding.

#### VAS questionnaires for appetite profile and palatability

Validated VAS questionnaires assessing hunger, fullness, satiation, desire to eat, and prospective food consumption were completed during each experimental aminoacidemia treatment [30]. The VAS questionnaire has previously been described, and each question consisted of a 100-mm horizontal line rating scale [30,31]. The most positive and most negative ratings were anchored at each end of the line. The VAS questions included the following: *1*) How hungry are you? (Response range: “not at all” to “as hungry as I have ever felt”); *2*) How full are you? (Range: “not at all” to “as full as i have ever felt”); *3*) How satiated are you? (Range: “not at all” to “extremely”); *4*) How strong is your desire to eat? (Range: “very weak” to “very strong”), and *5*) How much do you think you could (or would want to) eat right now? (Range: “nothing at all” to “a very large amount”). During each treatment, participants received the same thorough instructions from one of the study investigators on the meaning of each appetite sensation, and how to rate their appetite sensations using a VAS. Participants were instructed to rate themselves by marking the point that was most appropriate to their feeling at that time. The distance from the point marked on the VAS to the left end of the scale was measured in millimeters.

A palatability VAS questionnaire (PQ) was used to assess the palatability of the WPC test beverages with P3 or placebo, as well as the ad libitum meals. The PQ included the following: *1*) visual appeal (response range: good to bad); *2*) smell (range: good to bad); *3*) taste (range: good to bad); *4*) aftertaste (range: much to none), and *5*) palatability (range: good to bad).

#### Ad libitum meal

The ad libitum meal weighing ∼1 kg was prepared via a study team member (YH) before each experimental treatment. This consisted of spaghetti (Barilla) with tomato and basil pasta sauce (Classico) and marble cheddar cheese (Black Diamond Cheese Limited). The meal was based on a previous research study evaluating the reproducibility of subjective appetite ratings and ad libitum test meals [32]. The ad libitum meal contained ∼562 kJ/100 g, with 20% of energy from protein, 65% of energy from carbohydrate, and 15% of energy from fat. The participants were instructed to eat as much or as little as desired until feeling comfortably full within 30 min. The meal was provided on a glass pan along with a personal plate with utensils. The meal was weighed before consumption and remaining contents were covertly weighed after the meal to calculate the energy intake. The ad libitum meal was consumed by the participant in isolation in a separate room without additional water or access to time cues, cell phones, or interactions with study team members.

#### Biochemical assessments

##### Plasma glucose and insulin

Plasma glucose and insulin concentrations were measured in duplicate via enzyme-linked immunosorbent assays using commercially available kits (glucose; catalog No. EIAGLUC; ALPCO; insulin: catalog No. 80-INSHU-E10.1; Invitrogen by Fisher Scientific, Life Technology). The glucose kit had a reported concentration range of 36.7–246.7 mg/dL and sensitivity of 0.413 mg/dL. The mean percent coefficient of variation (CV) for the plasma glucose samples was 3.8%. The insulin kit had a reported concentration range of 3.0–200 μIU/mL and sensitivity of 0.399 μIU/mL. The mean percent CV for the plasma insulin samples was 5.4%.

##### Plasma orexigenic and anorexigenic hormones

Plasma active GLP-1, total PYY, and active ghrelin concentrations were measured in duplicate by multiplex immunoassays using a MESO QuickPlex SQ 120MM custom kit (Meso Scale Diagnostics). The active GLP-1 assay had a reported concentration range of 0.014–57 pM and sensitivity of 0.014 pM. The mean percent CV for the plasma active GLP-1 samples was 2.6%. The total PYY assay had a concentration range of 2.7–2260 pg/mL and sensitivity of 2.7 pg/mL. The mean percent CV for the plasma total PYY samples was 2.3%. The active ghrelin assay had a reported concentration range of 13–7160 pg/mL and sensitivity of 13 pg/mL. The mean percent CV for the plasma active ghrelin samples was 3.6%.

##### Plasma amino acids

Plasma amino acid concentrations were assessed in collaboration with the Proteomics and Molecular Analysis platform at the Research Institute of the McGill University Health Centre. Amino acids were extracted from plasma using protein precipitation and derivatized with 6-aminoquinolyl-N-hydroxysuccinimidyl carbamate (Toronto Research Chemicals) for analysis using reversed phase ultraperformance liquid chromatography mass spectrometry (UPLC-MS). Plasma samples were extracted alongside a calibration curve of amino acids in 0.1 N HCl with norvaline as an internal standard (all amino acids and norvaline purchased from Sigma-Aldrich). A calibration curve of 5–1000 μM was used for all amino acids except cystine (2.5–500 μM). An internal standard working solution containing 50 μM norvaline in 5% of 5-sulfosalicylic acid was used to extract plasma and calibration samples. Internal standard working solution aliquots (50 μL) were added to sample aliquots (25 μL) in microcentrifuge tubes, vortexed and centrifuged at 10,000 × *g* at 10 °C for 15 min. Supernatant aliquots (10 μL) were transferred into glass tubes containing 70 μL buffer solution (0.2 M sodium borate, pH 8.8) along with 20 μL derivatization solution (10 mM of 6-aminoquinolyl-N-hydroxysuccinimidyl carbamate in acetonitrile), mixed, and incubated for 10 min at 55 °C. After cooling to room temperature, aliquots (10 μL) were transferred to autosampler vials containing 1000 μL of type 1 water for UPLC-MS analysis.

Extracts were analyzed by UPLC-MS using an Agilent 6460 triple quadrupole mass spectrometer coupled with an Agilent 1290 UPLC system (Agilent Technologies). Extracts (10 μL) were injected onto an Agilent Eclipse Plus C18 100 × 2.1 mm (1.8 μm) column and chromatographed with a reverse phase gradient at 0.200 mL/min using 0.1% formic acid in water and 0.1% formic acid in acetonitrile. The derivatized amino acids were detected using electrospray positive mode ionization followed by MS/MS fragmentation. Data acquisition was performed using Agilent MassHunter Data Acquisition software (version B.05.00). Peak area measurements from selected product ions, calibration curve regression analysis and resulting sample quantification were performed using Agilent MassHunter Quantitative Analysis software (version B.05.00 SP02).

### Outcomes

The primary outcome variable was 120-min iAUC for postprandial plasma TAA concentration (μmol·L^−1^·120 min). All other outcomes were secondary outcome variables, except the 60-min iAUC for postprandial plasma amino acid concentration (μmol·L^−1^·60 min), which was a post hoc outcome variable.

### Statistical analyses

#### In vitro digestion simulations

In vitro simulation outcomes (TAA concentration, EAA concentration, BCAA concentration, leucine concentration, and FAN concentration) are reported as mean and SD. Separate 1-way analysis of variance was performed for each of these outcomes to compare means between the NC, PP, P3, and PP+P3 groups. Model assumptions were investigated using analysis of residuals. Post hoc pairwise comparisons were assessed when warranted, and *P* values were adjusted for multiple testing with the Tukey Honestly Significant Difference (HSD) test. All hypothesis tests were 2 sided and performed at the α = 0.05 significance level. Statistical analyses were performed, and figures were generated with GraphPad Prism version 10.1.0 for Windows (GraphPad Software).

#### Clinical trial: efficacy and ancillary assessments

Participant characteristics were summarized using descriptive statistics (mean and SD). Maximum concentration (*C*_max_) and time of maximum concentration (*T*_max_) for plasma amino acids were compared between treatments via analysis of covariance (ANCOVA) models. Specifically, a mixed-model approach was applied, adjusting for the covariates treatment period (i.e., treatment visit 1 and treatment visit 2), treatment sequence (i.e., P3 then placebo or placebo then P3), and sex (male or female). The mixed model allows for considering the within-subject correlated errors, since each subject received both treatments, by adding a random effect for each subject [33]. The iAUC for plasma glucose, insulin, amino acids, appetite regulatory hormones, and VAS-derived appetite sensations was calculated by trapezoidal integration as described and adapted from Brouns et al. [34]. The iAUC and ad libitum energy intake data was compared between treatments using a mixed-model ANCOVA, adjusting for the covariates treatment period, treatment sequence, and sex. Time–response data were analyzed via a mixed-model repeated measures approach adjusting for the covariates baseline values (i.e., *t* = 0 min measured before each treatment intake), treatment period, treatment sequence, and sex. The difference between the 2 baseline values was used as a covariate in the model [35]. We were a priori interested in comparing treatment means for these data. In cases where there was no interaction effect between treatment and time, we checked for a treatment effect and tested for a difference in treatment means. In cases where there was an interaction effect, we proceeded to compare treatment means at each time point, and *P* values were adjusted for multiple testing using the Bonferroni method. In all ANCOVA models, assumptions regarding the residuals—specifically, homogeneity of variance and normality—as well as the presence of influential observations (i.e., outliers) were investigated with graphical analysis of residuals. When residuals did not show evidence of following a normal (Gaussian) distribution and/or homogeneity of variance, values of the dependent variable were log transformed (natural logarithm) to ensure data followed an approximately normal distribution and/or to stabilize the variance. All hypothesis tests were 2 sided and performed at the α = 0.05 significance level. Results presented in table format (except participant characteristics) and text are reported as covariate-adjusted mean, SE, difference in covariate-adjusted mean, and 95% CI for the difference in covariate-adjusted means. In any model where the outcome was log transformed, the covariate-adjusted mean estimates were backtransformed by exponentiating them. To compare treatment groups, the ratio of adjusted geometric means and 95% CI is reported. Data presented in figure format are reported as unadjusted mean and SD. All statistical analyses were performed using the lme4 package [36] of R software [37] according to the statistical analysis plan outlined in the study protocol.

## Results

### In vitro digestion simulations

**FIGURE 2:**
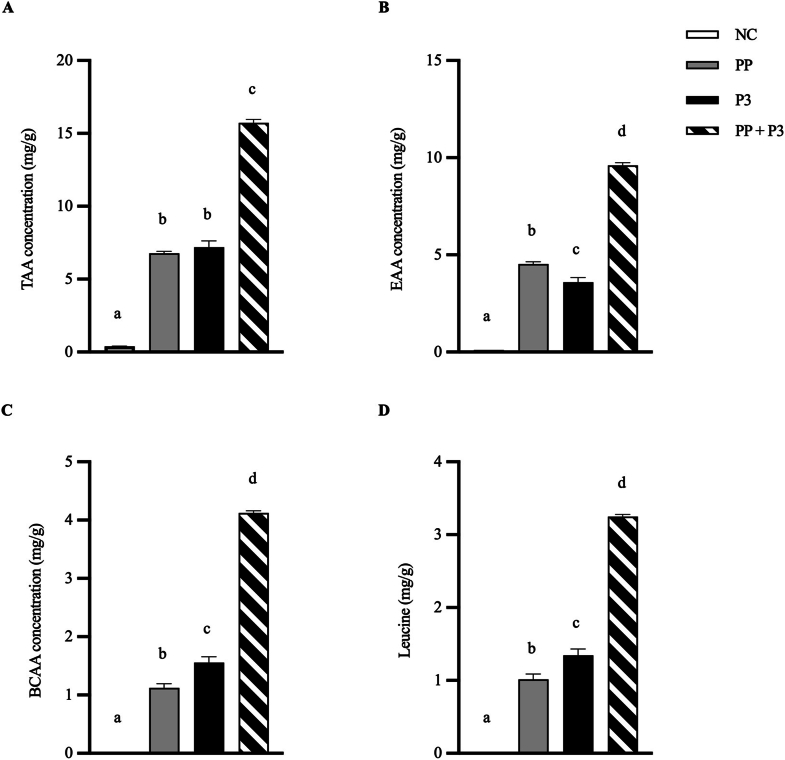
Mean gastric digesta of (A) TAA, (B) total EAA, (C) total BCAA, and (D) leucine concentrations after orogastric simulation of whey protein concentrate (WPC) digestion with and without P3 treatment. Results are reported in milligrams of amino acids per gram of WPC. Error bars show ±1 SD. Simulations were performed in triplicate. Significant differences between groups (*P* < 0.05) are denoted by unshared lowercase letters (a, b, c, and d). BCAA, branched-chain amino acid; EAA, essential amino acid; NC, negative control (no enzymes, WPC only); P3, mixture of 3 microbial protease preparations; PP, porcine pepsin (added to simulated gastric fluid); TAA, total amino acid.

**FIGURE 3:**
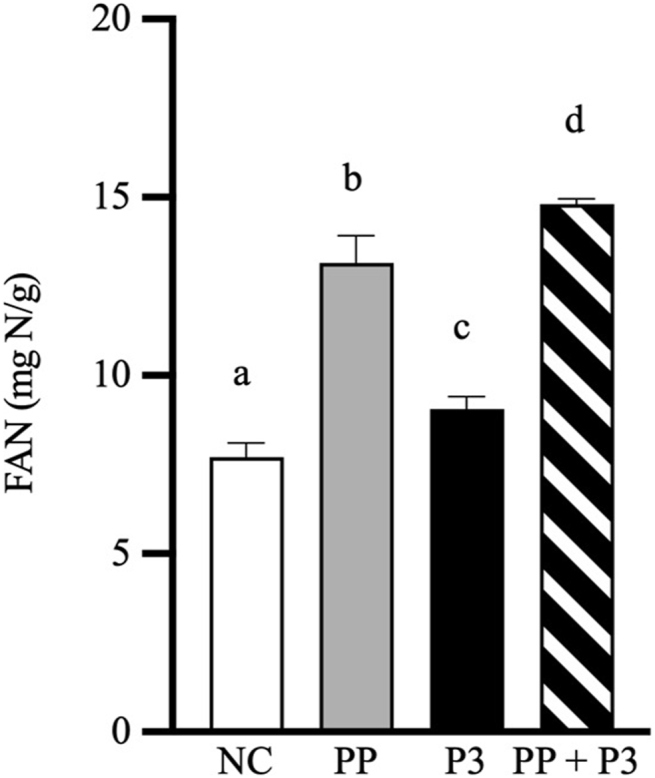
Mean gastric digesta FAN concentrations after orogastric simulation of whey protein concentrate (WPC) digestion with and without P3 treatment. Results are reported in milligrams of nitrogen per gram of WPC. Error bars show ±1 SD. Simulations were performed in triplicate. Significant differences between groups (*P* < 0.05) are denoted by unshared lowercase letters (a, b, c, d). FAN, free amino nitrogen; NC, negative control (no enzymes, WPC only); P3, mixture of 3 microbial protease preparations; PP, porcine pepsin (added to simulated gastric fluid).

The proteolytic efficacy of P3 at a fractional dose equivalent to 50,000 HUT per 31.9 g serving of WPC was confirmed in vitro. After orogastric simulation of WPC digestion with PP and P3, gastric digesta TAA, EAA, BCAA, and leucine concentrations increased by 132%, 112%, 267%, and 220%, respectively, compared with PP alone (all *P* < 0.0001) (Figure 2). Evaluation of P3 without PP showed a similar exoproteolytic pattern to PP alone with respect to TAA, despite small differences in EAA, BCAA, and leucine subcategories. The NC shows the inability of acid hydrolysis alone to release free amino acids. Importantly, P3 activity is not reduced by PP in the simulation, suggesting that PP does not digest or inhibit the proteases in P3. Moreover, PP and P3 complement each other to additively enhance exopeptidase activity compared with either alone. Confirmatory testing of release of FAN associated with both peptides and amino acids showed that average gastric digesta FAN concentration increased by 12.5% with P3 compared with that by PP alone (*P* = 0.012) (Figure 3). Incremental benefit of P3 on FAN release compared with amino acid release was expected to be less because PP typically shows robust endoprotease activity and little exopeptidase activity [19].

### Clinical trial

#### Participant characteristics

**FIGURE 4:**
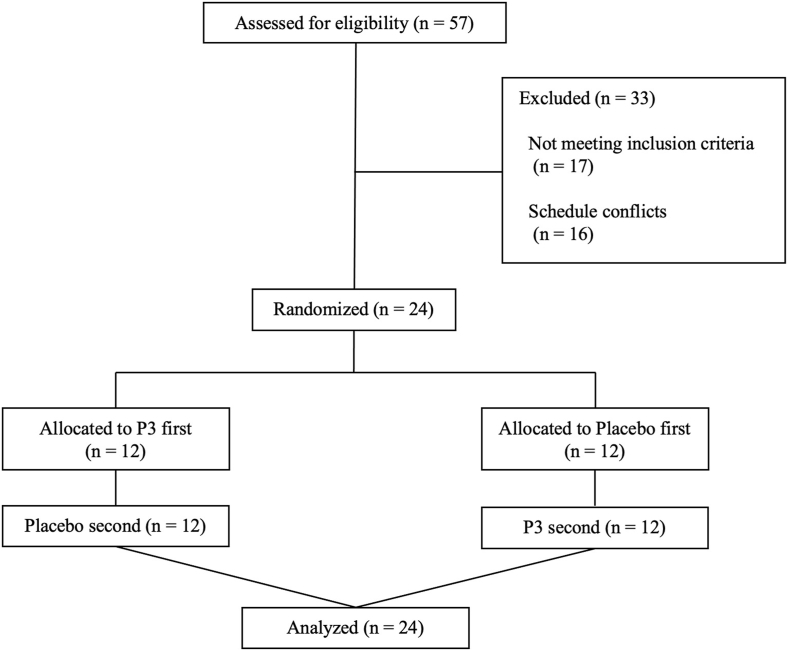
CONSORT flow diagram of participant screening and allocation to start with P3 or placebo in a crossover clinical trial. P3, whey protein beverage containing the mixture of 3 microbial protease preparations; placebo, whey protein beverage containing placebo.

All 24 participants [age—mean: 25 y (SD: 3 y); BMI—mean: 23.6 (SD: 2.9)] who were randomly assigned completed both treatments. Participants’ baseline characteristics and dietary intake are shown in Table 2. The CONSORT flow diagram of the study participants is shown in Figure 4.

**TABLE 2:** Baseline characteristics of study participants who ingested nutritional treatments consisting of whey protein concentrate with P3 or placebo.

Characteristic	Total (*N* = 24)	Female (*n* = 12)	Male (*n* = 12)
Age (y)	25 (3)	24 (3)	25 (3)
Height (cm)	168.3 (9.9)	161.7 (5.4)	174.8 (9.1)
Weight (kg)	67.1 (12.7)	59.3 (5.2)	74.9 (13.4)
BMI (kg/m^2^)	23.6 (2.9)	22.8 (2.9)	24.3 (2.9)
Systolic blood pressure (mm Hg)	111 (11)	103 (9)	118 (7)
Diastolic blood pressure (mm Hg)	74 (8)	73 (7)	75 (8)
Body fat (%)	26.4 (6.7)	31.6 (6.7)	21.2 (7.9)
Lean body mass (kg)	47.4 (11.3)	38.8 (4.8)	56.0 (8.9)
Fasting glucose (mmol/L)	4.24 (0.59)	4.27 (0.57)	4.20 (0.63)
Energy Intake (kj /d)	7991 (2071)	7165 (1813)	8816 (2047)
Relative protein intake (g/kg/d)	1.30 (0.61)	1.29 (0.61)	1.32 (0.52)
Protein intake (g/d)	87 (41)	76 (41)	98 (39)
Carbohydrate intake (g/d)	218 (54)	212 (55)	224 (55)
Fat intake (g/d)	79 (24)	68 (21)	89 (23)
TFEQ-R18 cognitive restraint	12.7 (3.8)	13.9 (3.1)	11.4 (4.2)
TFEQ-R18 uncontrolled eating	14.6 (3.6)	14.7 (4.1)	14.5 (3.2)
TFEQ-R18 emotional eating	5.2 (1.9)	5.0 (1.8)	5.3 (2.1)

Values represent mean (SD). Energy and macronutrient intake were determined from the 2-d dietary log.

Abbreviations: P3, whey protein beverage containing the mixture of 3 microbial protease preparations; TFEQ-R18, 3-Factor Eating Questionnaire Revised 18-item version.

#### Plasma amino acid concentration

**FIGURE 5:**
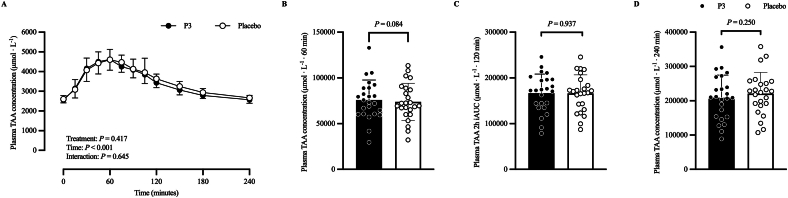
Plasma TAA (A) concentration at baseline (*t* = 0 min) and postprandial time points (*t* = 15‒240 min) and corresponding 60-min (B) iAUC (μmol·L^−1^·60 min), 120-min (C) iAUC (μmol·L^−1^·120 min), and 240-min (D) iAUC (μmol·L^−1^·240 min) of participants who consumed whey protein with P3 or placebo. Values represent unadjusted mean ± 1 SD (*N* = 24 participants). Time–response data were analyzed via a mixed-model repeated-measures analysis of covariance (ANCOVA) adjusted for baseline values, treatment period, treatment sequence, and sex. iAUC data were analyzed via a mixed-model ANCOVA adjusted for treatment period, treatment sequence, and sex. iAUC, incremental area under the curve; P3, whey protein beverage containing the mixture of 3 microbial protease preparations; placebo, whey protein beverage containing placebo; TAA, total amino acid.

Figure 5 shows the plasma TAA (Figure 5A) concentration (in micromoles per liter) time–response profile, 60-min (Figure 5B) iAUC (μmol·L^−1^·60 min), 120-min (Figure 5C) iAUC (μmol·L^−1^·120 min), and 240-min (Figure 5D) iAUC (μmol·L^−1^·240 min). The adjusted mean postprandial plasma TAA concentration (μmol·L^−1^) time–response profile increased after protein ingestion (time: *P* < 0.001), with no difference between treatments (*P* = 0.417) or treatment × time interaction (*P* = 0.645). Adjusted geometric mean postprandial TAA 60-min iAUC (μmol·L^−1^·60 min) was 75,461 (SE: 4740) and 68,231 (SE: 4290) in the P3 and placebo treatment, respectively. Their ratio was not statistically significantly different from the null value of 1 (ratio: 1.11; 95% CI: 0.99, 1.24; *P* = 0.084). Adjusted mean postprandial TAA 120-min iAUC (μmol·L^−1^·120 min) was 166,610 (SE: 8020) and 166,073 (SE: 8020) in the P3 and placebo treatments, respectively, and was not significantly different between treatments (difference: −537; 95% CI: −13,973, 12,899; *P* = 0.937). Adjusted mean postprandial TAA 240-min iAUC (μmol·L^−1^·240 min) was 208,717 (SE: 11,277) and 220,878 (SE: 11,277) in the P3 and placebo treatments, respectively, and was not significantly different between treatments (difference: 12161; 95% CI: −8437, 32,759; *P* = 0.250).

**FIGURE 6:**
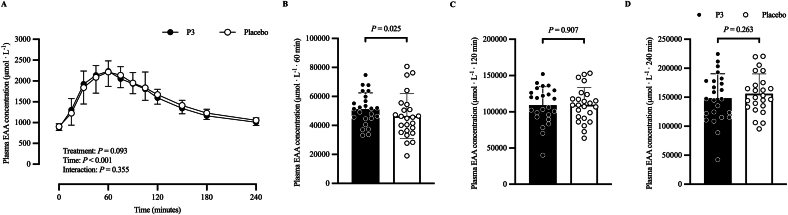
Plasma EAA (A) concentration at baseline (*t* = 0 min) and postprandial time points (*t* = 15‒240 min) and corresponding 60-min (B) iAUC (μmol·L^−1^·60 min), 120-min (C) iAUC (μmol·L^−1^·120 min), and 240-min (D) iAUC (μmol·L^−1^·240 min) of participants who consumed whey protein with P3 or placebo. Values represent unadjusted mean ± 1 SD (*N* = 24 participants). Time–response data were analyzed via a mixed-model repeated-measures analysis of covariance (ANCOVA) adjusted for baseline values, treatment period, treatment sequence, and sex. iAUC data were analyzed via a mixed-model ANCOVA adjusted for treatment period, treatment sequence, and sex. EAA, essential amino acid; iAUC, incremental area under the curve; P3, whey protein beverage containing the mixture of 3 microbial protease preparations; placebo, whey protein beverage containing placebo.

Figure 6 shows the plasma EAA (Figure 6A) concentration (in micromoles per liter) time–response profile, 60-min (Figure 6B) iAUC (μmol·L^−1^·60 min), 120-min (Figure 6C) iAUC (μmol·L^−1^·120 min), and 240-min (Figure 6D) iAUC (μmol·L^−1^·240 min). The adjusted mean postprandial plasma EAA concentration (μmol/L) time–response profile increased after protein ingestion (time: *P* < 0.001), with no difference between treatments (*P* = 0.093) or treatment × time interaction (*P* = 0.355). Adjusted geometric mean postprandial EAA 60-min iAUC (μmol·L^−1^·60 min) was 50,149 (SE: 2940) and 44,001 (SE: 2580) in the P3 and placebo treatments, respectively. Their ratio was statistically significantly different from the null value of 1 (ratio: 1.14; 95% CI: 1.02, 1.28; *P* = 0.025). Adjusted mean postprandial plasma EAA 120-min iAUC (μmol·L^−1^·120 min) was 109,028 (SE: 5107) and 109,535 (SE: 5107) in the P3 and placebo treatments, respectively, and was not significantly different between treatments (difference: 507; 95% CI: −8099, 9113; *P* = 0.907). Adjusted mean postprandial plasma EAA 240-min iAUC (μmol·L^−1^·240 min) was 148,367 (SE: 7455) and 156,059 (SE: 7455) in the P3 and placebo treatments, respectively, and was not significantly different between treatments (difference: 7692; 95% CI: −5698, 21,082; *P* = 0.263).

**FIGURE 7:**
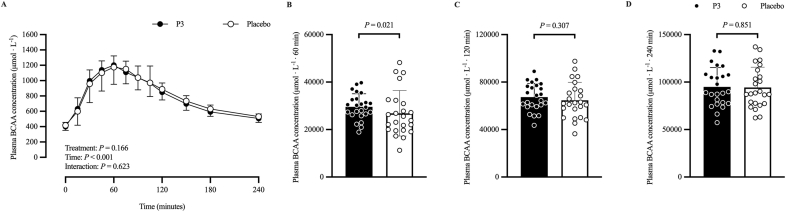
Plasma BCAA (A) concentration at baseline (*t* = 0 min) and postprandial time points (*t* = 15‒240 min) and corresponding 60-min (B) iAUC (μmol·L^−1^·60 min), 120-min (C) iAUC (μmol·L^−1^·120 min), and 240-min (D) iAUC (μmol·L^−1^·240 min) of participants who consumed whey protein with P3 or placebo. Values represent unadjusted mean ± 1 SD (*N* = 24 participants). Time–response data were analyzed via a mixed-model repeated-measures analysis of covariance (ANCOVA) adjusted for baseline values, treatment period, treatment sequence, and sex. iAUC data were analyzed via a mixed-model ANCOVA adjusted for treatment period, treatment sequence, and sex. BCAA, branched-chain amino acid; iAUC, incremental area under the curve; P3, whey protein beverage containing the mixture of 3 microbial protease preparations; placebo, whey protein beverage containing placebo.

Figure 7 shows the plasma BCAA (Figure 7A) concentration (in micromoles per liter) time–response profile, 60-min (Figure 7B) iAUC (μmol·L^−1^·60 min), 120-min (Figure 7C) iAUC (μmol·L^−1^·120 min), and 240-min (Figure 7D) iAUC (μmol·L^−1^·240 min). The adjusted mean postprandial plasma BCAA concentration (μmol/L) time–response profile increased after protein ingestion (time: *P* < 0.001), with no difference between treatments (*P* = 0.166) or treatment × time interaction (*P* = 0.623). Adjusted geometric mean postprandial plasma BCAA 60-min iAUC (μmol·L^−1^·60 min) was 29,080 (SE: 1680) and 25,314 (SE: 1460) in the P3 and placebo treatments, respectively. Their ratio was statistically significantly different from the null value of 1 (ratio: 1.15; 95% CI: 1.02, 1.29; *P* = 0.021). Adjusted mean postprandial plasma BCAA 120-min iAUC (μmol·L^−1^·120 min) was 67,303 (SE: 2743) and 64,695 (SE: 2743) in the P3 and placebo treatments, respectively, and was not significantly different between treatments (difference: −2608; 95% CI: −7590, 2374; *P* = 0.307). Adjusted mean postprandial plasma BCAA 240-min iAUC (μmol·L^−1^·240 min) was 94,892 (SE: 3959) and 94,269 (SE: 3959) in the P3 and placebo treatments, respectively, and was not significantly different between treatments (difference: −623; 95% CI: −7169, 5923; *P* = 0.851).

**FIGURE 8:**
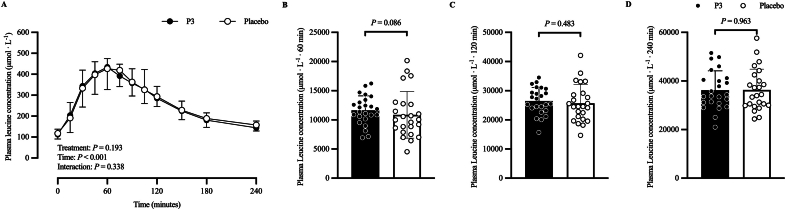
Plasma leucine (A) concentration at baseline (*t* = 0 min) and postprandial time points (*t* = 15‒240 min) and corresponding 60-min (B) iAUC (μmol·L^−1^·60 min), 120-min (C) iAUC (μmol·L^−1^·120 min), and 240-min (D) iAUC (μmol·L^−1^·240 min) of participants who consumed whey protein with P3 or placebo. Values represent unadjusted mean ± 1 SD (*N* = 24 participants). Time–response data were analyzed via a mixed-model repeated-measures analysis of covariance (ANCOVA) adjusted for baseline values, treatment period, treatment sequence, and sex. iAUC data were analyzed via a mixed-model ANCOVA adjusted for treatment period, treatment sequence, and sex. P3, whey protein beverage containing the mixture of 3 microbial protease preparations; placebo, whey protein beverage containing placebo.

Figure 8 shows the plasma leucine (Figure 8A) concentration in micromoles per liter) time–response profile, 60-min (Figure 8B) iAUC (μmol·L^−1^·60 min), 120-min (Figure 8C) iAUC (μmol·L^−1^·120 min), and 240-min (Figure 8D) iAUC (μmol·L^−1^·240 min). The adjusted mean postprandial plasma leucine concentration (μmol/L) time–response profile increased after protein ingestion (time: *P* < 0.001), with no difference between treatments (*P* = 0.193) or treatment × time interaction (*P* = 0.338). Adjusted geometric mean postprandial plasma leucine 60-min iAUC (μmol·L^−1^·60 min) was 11,373 (SE: 697) and 10,223 (SE: 626) in the P3 and placebo treatments, respectively. Their ratio was not statistically significantly different from the null value of 1 (ratio: 1.11; 95% CI: 0.98, 1.26; *P* = 0.086). Adjusted mean postprandial plasma leucine 120-min iAUC (μmol·L^−1^·120 min) was 26,481 (SE: 1149) and 25,690 (SE: 1149) in the P3 and placebo treatments, respectively, and was not significantly different between treatments (difference: −791; 95% CI: −3007, 1425; *P* = 0.483). Adjusted mean postprandial plasma leucine 240-min iAUC (μmol·L^−1^·240 min) was 36,244 (SE: 1623) and 36,313 (SE: 1623) in the P3 and placebo treatments, respectively, and was not significantly different between treatments (difference: 69; 95% CI: −2815, 2953; *P* = 0.963).

Adjusted mean *C*_max_ and *T*_max_, SE, mean difference, 95% CI of the difference, and corresponding *P* value for plasma TAA, EAA, BCAA, and leucine are shown in Table 3. There were no treatment-dependent differences for any of these outcome measures.

**TABLE 3:** *C*_max_ and *T*_max_ for postprandial plasma TAA, EAA, BCAA, and leucine of study participants who consumed whey protein concentrate with P3 or placebo.

	*C*_max_ (μmol/L)				*T*_max_ (min)			
	P3	Placebo	Difference (95% CI)	*P*	P3	Placebo	Difference (95% CI)	*P*
TAA	4808 (114)	4746 (114)	−62 (−298, 174)	0.601	57 (4)	64 (4)	7 (−5, 19)	0.188
EAA	2310 (60)	2290 (60)	−20 (−146, 106)	0.754	58 (4)	66 (4)	8 (−2, 18)	0.118
BCAA	1253 (35)	1236 (35)	−17 (−87, 53)	0.625	58 (4)	68 (4)	10 (0, 20)	0.063
Leucine	458 (13)	452 (13)	−6 (−34, 22)	0.647	60 (4)	67 (4)	7 (−3, 17)	0.218

Maximum plasma TAA, EAA, BCAA, and leucine concentrations and time of maximum concentrations during postprandial conditions (*t* = 15–240 min) after protein beverage intake in young adults. Values represent adjusted mean, SE, mean difference, and 95% CI of the difference. *N* = 24 study participants. All data were analyzed via a mixed-model analysis of covariance adjusted for treatment period, treatment sequence, and sex.

Abbreviations: BCAA, branched-chain amino acid; *C*_max_, maximum concentration; EAA, essential amino acid; P3, whey protein beverage containing the mixture of 3 microbial protease preparations; placebo, whey protein beverage containing placebo; TAA, total amino acid; *T*_max_, time of maximum concentration.

#### Plasma glucose and insulin concentrations

**FIGURE 9:**
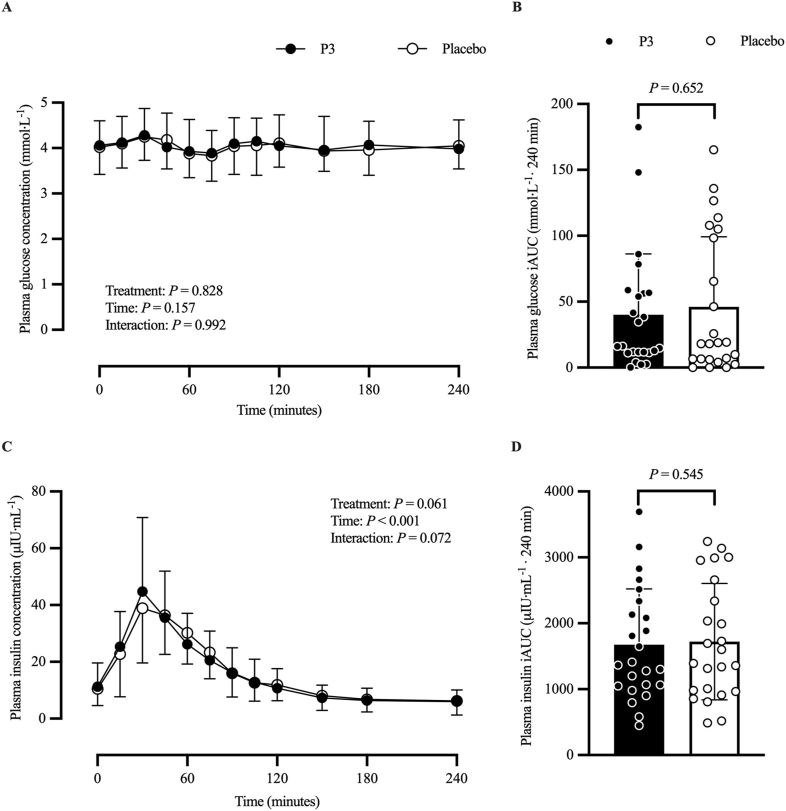
Plasma glucose (A) and insulin (C) concentrations at baseline (*t* = 0 min) and postprandial time points (*t* = 15‒240 min) and corresponding iAUC for glucose (B; mmol·L^−1^·240 min) and insulin (D; μIU·mL^−1^·240 min) of participants who consumed whey protein with P3 or placebo. Values represent unadjusted mean ± 1 SD (*N* = 24 participants). Time–response data were analyzed via a mixed-model repeated-measures analysis of covariance (ANCOVA) adjusted for baseline values, treatment period, treatment sequence, and sex. iAUC data were analyzed via a mixed-model ANCOVA adjusted for treatment period, treatment sequence, and sex. iAUC, incremental area under the curve; P3, whey protein beverage containing the mixture of 3 microbial protease preparations; placebo, whey protein beverage containing placebo.

Figure 9 shows plasma glucose (Figure 9A, B) and insulin (Figure 9C, D) concentrations. The adjusted mean postprandial plasma glucose concentration (in millimoles per liter) time–response profile did not differ between treatments. Adjusted mean postprandial plasma glucose iAUC (mmol·L^−1^·240 min) was 40.1 (SE: 10.3) and 46.1 (SE: 10.3) in the P3 and placebo treatments, respectively, and was not significantly different between treatments (difference: 6; 95% CI: −20.6, 32.6; *P* = 0.652). The adjusted mean log-transformed postprandial plasma insulin concentration (in μIU/mL) time–response profile showed a main effect of time (*P* < 0.001) but did not differ between treatments (*P* = 0.061). Adjusted mean postprandial plasma insulin iAUC (μIU·mL^−1^·240 min) was 1675 (SE: 165) and 1722 (SE: 165) in the P3 and placebo treatments, respectively, and was not significantly different between treatments (difference: 47; 95% CI: −105, 199; *P* = 0.545).

#### Plasma active ghrelin, active GLP-1, and total PYY concentrations

**FIGURE 10:**
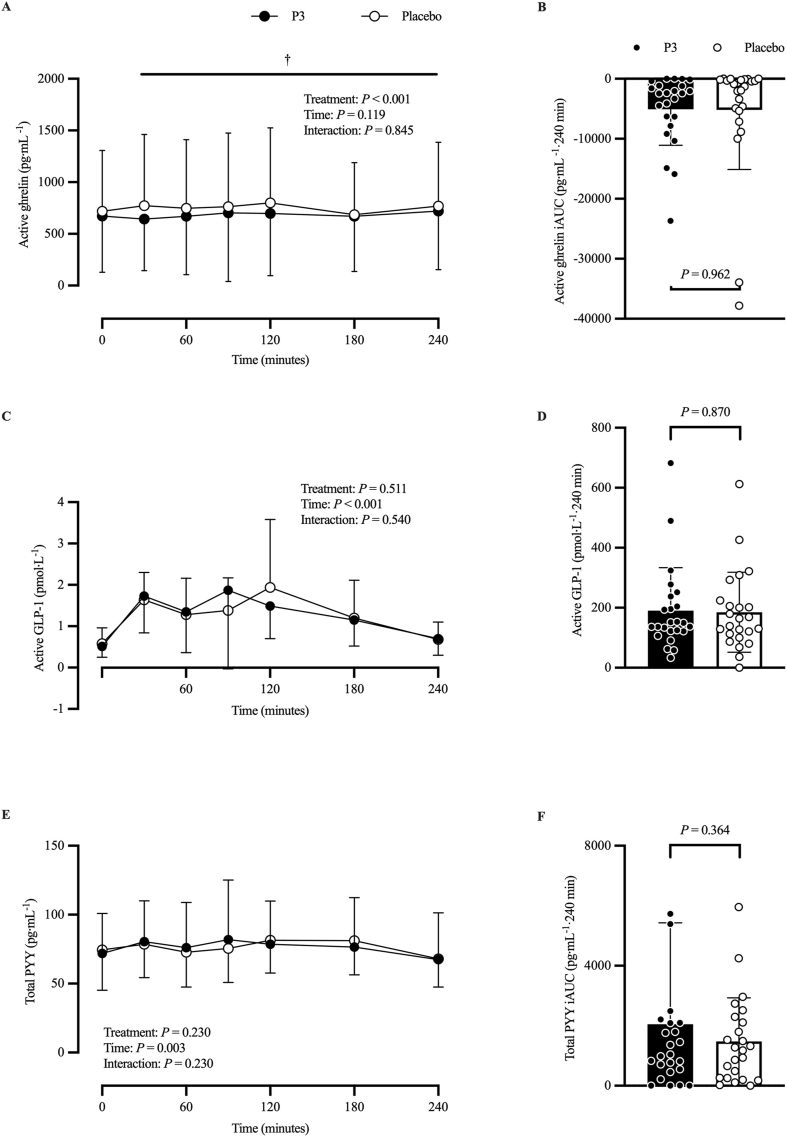
Plasma active ghrelin (A), active GLP-1 (C), and total PYY (E) concentrations at baseline (*t* = 0 min) and postprandial time points (*t* = 30‒240 min) and corresponding iAUC for active ghrelin (B; pg·mL^−1^·240 min), active GLP-1 (D; pmol·L^−1^·240 min), and total PYY (F, pg·mL^−1^·240 min) of participants who consumed whey protein with P3 or placebo. Values represent unadjusted mean ± 1 SD. Time–response data were analyzed via a mixed-model repeated-measures analysis of covariance (ANCOVA) adjusted for baseline values, treatment period, treatment sequence, and sex. iAUC data were analyzed via a mixed-model ANCOVA adjusted for treatment period, treatment sequence, and sex. ^†^Difference between treatments, *P* < 0.05. GLP, glucagon-like peptide; iAUC, incremental area under the curve; P3, whey protein beverage containing the mixture of 3 microbial protease preparations; placebo, whey protein beverage containing placebo; PYY, peptide YY.

Figure 10 shows plasma active ghrelin (Figure 10A, B), active GLP-1 (Figure 10C, D), and total PYY (Figure 10E, F) concentration. The model for log-transformed postprandial plasma active ghrelin concentration (in picograms per milliliter) time–response profile showed no significant treatment × time interaction (*P* = 0.845). Adjusted geometric mean plasma active ghrelin concentration (in picograms per milliliter) was 394 (SE: 43.1) and 449 (SE: 49.1) in the P3 and placebo treatments, respectively. Their ratio was statistically significantly different from the null value of 1 (ratio: 0.88; 95% CI: 0.83, 0.94; *P* < 0.001). Adjusted mean postprandial plasma active ghrelin iAUC (pg·mL^−1^·240 min) was −5091 (SE: 1640) and −5183 (SE: 1640) in the P3 and placebo treatments, respectively, and was not significantly different between treatments (difference: 92; 95% CI: −3809, 3625; *P* = 0.962).

Adjusted mean postprandial plasma active GLP-1 concentration (in picomoles per liter) time–response profile demonstrated a main effect of time (time: *P* < 0.001), but no difference between treatments (*P* = 0.511) or treatment × time interaction (*P* = 0.540). Adjusted mean postprandial plasma active GLP-1 iAUC (pmol·L^−1^·240 min) was 191 (SE: 29) and 185 (SE: 29) in the P3 and placebo treatments, respectively, and was not significantly different between treatments (difference: −6; 95% CI: −74, 62; *P* = 0.870).

Adjusted mean postprandial plasma total PYY concentration (in picograms per milliliter) time–response profile demonstrated a main effect of time (time: *P* = 0.003), but no difference between treatments (*P* = 0.230) or treatment × time interaction (*P* = 0.230). Adjusted mean postprandial plasma total PYY iAUC (pg·mL^−1^·240 min) was 2047 (SE: 541) and 1475 (SE: 541) in the P3 and placebo treatments, respectively, and was not significantly different between treatments (difference: −572; 95% CI: −1806, 662; *P* = 0.364).

#### VAS-derived appetite sensations and ad libitum meal intake

**FIGURE 11:**
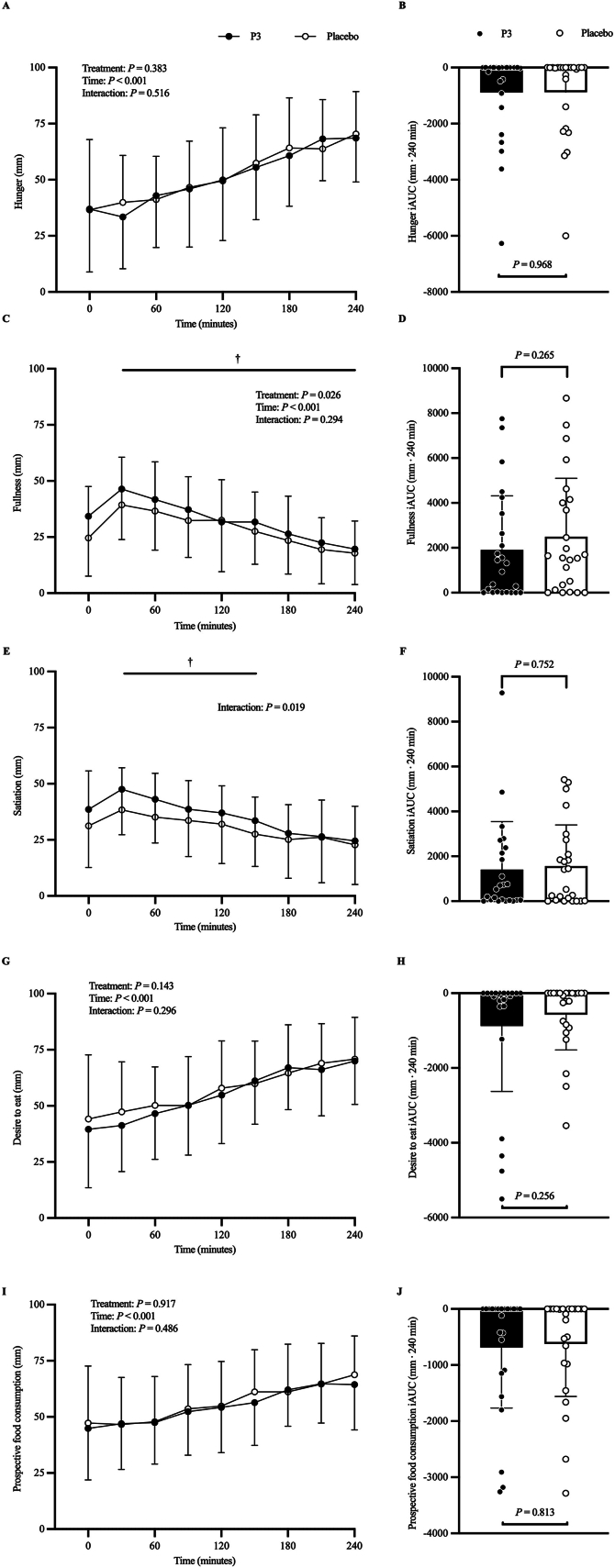
Visual analog scale–derived hunger (A), fullness (C), satiation (E), desire to eat (G), and prospective food consumption (I) at baseline (*t* = 0 min) and postprandial time points (*t* = 30‒240 min) and corresponding iAUC (mm·240 min) for hunger (B), fullness (D), satiation (F), desire to eat (H), and prospective food consumption (J) of participants who consumed whey protein with P3 or placebo. Values represent unadjusted mean ± 1 SD (*N* = 24 participants). Time–response data were analyzed via a mixed-model repeated-measures analysis of covariance (ANCOVA) adjusted for baseline values, treatment period, treatment sequence, and sex. iAUC data were analyzed via a mixed-model ANCOVA adjusted for treatment period, treatment sequence, and sex. Adjusted means between treatments were compared at each time point when there was a treatment × time interaction effect, and *P* values were adjusted for multiple testing. ^†^Difference between treatments, *P* < 0.05. iAUC, incremental area under the curve; P3, whey protein beverage containing the mixture of 3 microbial protease preparations; placebo, whey protein beverage containing placebo.

Figure 11 shows VAS-derived sensations of hunger (Figure 11A, B), fullness (Figure 11C, D), satiation (Figure 11E, F), desire to eat (Figure 11G, H), and prospective food consumption (Figure 11I, J). The adjusted mean postprandial hunger (in millimeters) time–response profile showed a main effect of time (*P* < 0.001), but no difference between treatments (*P* = 0.383) or treatment × time interaction (*P* = 0.516). Adjusted mean postprandial hunger iAUC (mm·240 min) below baseline was −890 (SE: 316) and −878 (SE: 316) in the P3 and placebo treatments, respectively, and was not significantly different between treatments (difference: 12; 95% CI: −554, 578; *P* = 0.968).

The adjusted mean postprandial fullness (in millimeters) time–response profile showed a main effect of time (*P* < 0.001) and a main effect of treatment (*P* = 0.026), whereby fullness was higher in the P3 compared to placebo treatment; however, there was no treatment × time interaction (*P* = 0.294). Adjusted mean postprandial fullness iAUC (mm·240 min) above baseline was 1909 (SE: 512) and 2494 (SE: 512) in the P3 and placebo treatments, respectively, and was not significantly different between treatments (difference: 585; 95% CI: −437, 1607; *P* = 0.265).

The adjusted mean postprandial satiation (in millimeters) time–response profile showed a significant treatment × time interaction, whereby satiation was greater in P3 than that in placebo from 30–150 min in the postprandial period (all *P* < 0.05). Owing to the interaction effect, differences between treatments between time points (i.e., difference-in-differences contrasts) were investigated. Results showed no statistically significant differences (all *P* > 0.05). Adjusted mean postprandial satiation iAUC (mm·240 min) above baseline was 1408 (SE: 406) and 1565 (SE: 406) in the P3 and placebo treatments respectively, and was not significantly different between treatments (difference: 157; 95% CI: −823, 1137; *P* = 0.752).

The adjusted mean postprandial desire to eat (in millimeters) time–response profile showed a main effect of time (*P* < 0.001), but no difference between treatments (*P* = 0.143) or treatment ′ time interaction (*P* = 0.296). Adjusted mean postprandial desire to eat iAUC (mm·240 min) below baseline was −880 (SE: 290) and −573 (SE: 290) min in the P3 and placebo treatments, respectively, and did not differ between treatments (difference: 307; 95% CI: −219, 833; *P* = 0.256).

The adjusted mean postprandial prospective food consumption (in millimeters) time–response profile showed a main effect of time (*P* < 0.001), but no difference between treatments (*P* = 0.917) or treatment × time interaction (*P* = 0.486). Adjusted mean postprandial prospective food consumption iAUC (mm·240 min) below baseline was −686 (SE: 204) and −623 (SE: 204) in the P3 and placebo treatments, respectively, and did not differ significantly between treatments (difference: 63; 95% CI: −466, 592; *P* = 0.813).

**FIGURE 12:**
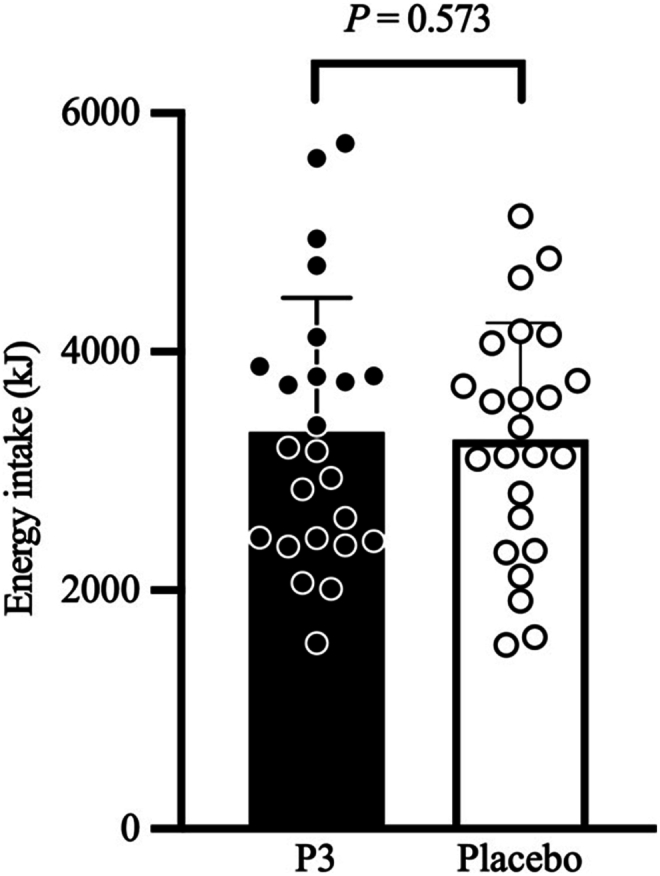
Ad libitum meal energy intake (kJ) 240 min after participants consumed whey protein with P3 or placebo. Values represent unadjusted mean ± 1 SD (*N* = 24 participants). Data for energy intake were analyzed via a mixed-model analysis of covariance adjusted for treatment period, treatment sequence, and sex. P3, whey protein beverage containing the mixture of 3 microbial protease preparations; placebo, whey protein beverage containing placebo.

Ad libitum meal energy intake is shown in Figure 12. Ad libitum meal energy intake (in kilojoules) 240 min after the ingestion of the P3 and placebo treatments was 3330 (SE: 189) and 3262 (SE: 189) kJ, respectively, and was not significantly different between treatments (difference: −68; 95% CI: −306, 170; *P* = 0.573).

#### VAS-derived palatability, mGITQ, and exit survey

Adjusted mean visual appearance was 36.6 (SE: 4.6) and 39.7 (SE: 4.6) mm (difference: 3.1; 95% CI: −6.5, 12.7; *P* = 0.531) in the P3 and placebo treatments, respectively. Adjusted mean smell was 44.5 (SE: 4.1) and 46.7 (SE: 4.1) mm (difference: 2.2; 95% CI: −4.8, 9.2; *P* = 0.533) in the P3 and placebo treatments, respectively. Adjusted mean taste was 49.0 (SE: 4.7) and 47.5 (SE: 4.7) mm (difference: −1.5; 95% CI: −9.3, 6.3; *P* = 0.706) in the P3 and placebo treatments, respectively. Adjusted mean aftertaste was 49.8 (SE: 5.3) in the P3 treatment and 61.0 (SE: 5.0) mm in the placebo treatment (difference: 11.2; 95% CI: 2.4, 20.0; *P* = 0.019) in which the higher the number, the less the aftertaste. Adjusted mean overall palatability was 42.9 (SE: 4.3) and 42.2 (SE: 4.3) mm in the P3 and placebo treatments, respectively (difference: −0.7; 95% CI: −10.1, 8.7; *P* = 0.875). Adjusted mean visual appearance of the ad libitum meal was 26.6 (SE: 3.9) and 22.3 (SE: 3.9) mm (difference: −4.3; 95% CI: −11.7, 3.1; *P* = 0.257) in the P3 and placebo treatments, respectively. Adjusted mean smell of the ad libitum meal was 26.1 (SE: 2.9) and 21.5 (SE: 2.9) mm (difference: −4.6; 95% CI: −11.0, 1.8; *P* = 0.162) in the P3 and placebo treatments, respectively. Adjusted mean taste of the ad libitum meal was 24.5 (SE: 3.2) and 22.1 (SE: 3.2) mm (difference: −2.4; 95% CI: −9.2, 4.4; *P* = 0.473) in the P3 and placebo treatments, respectively. Adjusted mean aftertaste of the ad libitum meal was 46.9 (SE: 6.0) and 51.5 (SE: 6.0) mm (difference: 4.6; 95% CI: −3.6, 12.8; *P* = 0.271) in the P3 and placebo treatments, respectively. Adjusted mean overall palatability of the ad libitum meal was 19.6 (SE: 2.7) and 18.1 (SE: 2.7) mm (difference: −1.5; 95% CI: −6.7, 3.7; *P* = 0.577) in the P3 and placebo treatments, respectively.

According to responses to the mGITQ, GI symptom severity was comparable between P3 and placebo treatments during the 240-min postprandial period. The 3-item severity sub-score was 20 and 16 in the P3 and placebo treatments, respectively. The 8-item total severity score was 41 and 37 in the P3 and placebo treatments, respectively.

At the end of their second study treatment visit, participants were asked what they thought their assigned treatment sequence was for the 2 treatment visits (i.e., P3 then placebo or placebo then P3). Of the 24 participants, 11 (46%) of the participants correctly guessed their randomly assigned treatment sequence. Of the participants who chose the correct sequence, their reported confidence was 2.4 of 5 in comparison with 2.2 of 5 in the participants who chose the incorrect sequence. The participants did not guess their treatment sequence with a level of accuracy much higher than would be expected with a random guess between 2 possible sequences (i.e., 50%).

## Discussion

In this study, we first demonstrated that P3 at 50,000 HUT per 31.9 g WPC can remarkably improve gastric release of amino acids in the in vitro digestion simulations. Subsequently, we conducted a randomized, double-blind, placebo-controlled crossover trial to assess the effect of co-ingestion of WPC with P3 compared with that of placebo on postprandial plasma amino acid, glucose, insulin, and appetite regulatory hormone concentrations, VAS-derived appetite sensations, ad libitum meal energy intake, and GI tolerability in healthy young adults. Our results demonstrate that P3 co-ingestion with WPC increased both postprandial plasma EAA and BCAA 60-min iAUC compared with placebo; however, there were no treatment differences for any other amino acid outcomes including the 60-min iAUC for TAA and leucine, nor the 120-min and 240-min iAUC, *C*_max_, *T*_max_, or their time–response profiles for TAA, EAA, BCAA, and leucine, respectively. The time–response profile data demonstrated that VAS-derived sensations of postprandial fullness and satiation were greater, whereas postprandial plasma active ghrelin concentration was lower with the P3 than that with placebo treatment. In terms of palatability, the P3 treatment had significantly more aftertaste than placebo; however, no other differences were observed between treatments.

There is emerging evidence that co-ingestion of microbial proteases with dietary protein may augment postprandial blood amino acid concentrations in humans [[20], [21], [22]]. Exogenous proteolytic enzyme supplementation may theoretically complement endogenous proteases (e.g., pepsin, trypsin, and chymotrypsin) already present in the digestive system to enhance the release of peptides and amino acids from ingested dietary protein. Results from both in vitro digestibility simulation studies [[15], [16], [17], [18], [19]], and in vivo human studies [[20], [21], [22]], suggest that proteolytic enzymes can further increase amino acid availability/concentration in response to dietary protein. For example, Paulussen et al. [22] recently compared postprandial plasma amino acid concentration following co-ingestion of 25 g pea protein isolate with a lower dose of P3 (31,875 HUT) with that of placebo in healthy young adults. Plasma TAA concentration increased over time, but with higher concentration reported for P3 than that for placebo during the 0‒5 h postprandial period. The early 0‒120 min, but not total 0‒300 min, iAUCs for leucine, BCAA, EAA, and TAA were also higher for the P3 than those for the placebo treatment [22]. Alternatively, Townsend et al. [23] reported no difference in postprandial plasma amino acid concentration in young males during recovery from an acute bout of resistance exercise following co-ingestion of 26 g WPC with 250 mg ProHydrolase (proteases from *Bacillus subtilis* and *Ananas comosus*) compared with a nonnutritive control. On the contrary, another human trial by Oben et al. [20] using 2.5 g and 5 g of Aminogen (proteases from *Aspergillus niger* and *A oryzae*) reported increased serum amino acid concentration following co-ingestion with 50 g WPC compared with that with 50 g WPC alone in young healthy male participants. Overall, these results suggest that the source, type, and/or dose of each of microbial protease and/or dietary protein (e.g., pea compared with whey protein) may influence the capacity of proteolytic enzymes to augment postprandial blood amino acid concentration. In this study, we compared the acute effects of ingesting 31.9 g WPC (containing 25 g total amino acids) with P3 with those of placebo on postprandial plasma amino acid concentration. Our findings revealed a greater EAA and BCAA 60-min iAUC for P3 than those for placebo; however, there were no treatment-dependent differences noted for the 60-min iAUC for TAA and leucine, nor the 120-min iAUC, 240-min iAUC, *C*_max_, *T*_max_, or time–response profiles for TAA, EAA, BCAA, and leucine, respectively. The subtlety of the effects of P3 co-ingestion with WPC on amino acid outcomes may relate to the fact that whey protein is already a high-quality protein source rich in EAA (typically ∼50%) that is rapidly digested and absorbed [38]. Whey protein is the most used supplemental protein source by athletes and sports nutrition product consumers to support skeletal muscle hypertrophy and improve body composition with exercise [39]. When compared with other sources of dietary protein, the Digestible Indispensable Amino Acid Score of WPC has been reported to be 133, a score substantially higher than reported for pea protein concentrate (73), soy protein isolate (98), and wheat (54) [40]. Although whey protein is already rapidly digested and absorbed, whey protein hydrolysate can further increase postprandial plasma amino acid concentration than whey [41,42]. Protein hydrolysates are produced by chemically unfolding proteins and enzymatically hydrolyzing peptide bonds at various points in the proteins primary structure to produce varying quantities of free amino acids and peptides [43]. Hydrolyzing a protein to create a protein hydrolysate essentially results in predigestion of the protein, which has been suggested to enhance amino acid absorption, resulting in a greater/more rapid increase in postprandial plasma amino acid concentrations [42,44,45]. The greater postprandial plasma EAA and BCAA 60-min iAUC with P3 than those with placebo treatment, yet absence of a treatment difference in *C*_max_ or *T*_max_ for amino acid outcomes (Table 3), may suggest that the microbial proteases present in P3 enhanced the capacity, rather than rapidity, of whey protein digestion/hydrolysis, thereby increasing initial (i.e., 60-min iAUC) amino acid availability in the circulation. Although protein hydrolysates can result in an accelerated digestion and absorption rate in vivo in humans when compared with intact versions of the same protein [45,46], proteases are more versatile. Protease co-ingestion has the potential to enhance the digestion of any protein within a meal setting, making them applicable in a broader range of real-world dietary contexts.

The plasma glucose response during the postprandial period did not vary over time and was not significantly different between treatments (Figure 9A). At the same time, plasma insulin concentration increased during the postprandial period but was not significantly different between treatments (Figure 9C). In healthy individuals, blood glucose concentration is tightly regulated to prevent both hypoglycemia and hyperglycemia, thereby ensuring normal body functions. Insulin is the key hormone responsible for lowering blood glucose concentration in the postprandial period following meal intake. Studies evaluating the insulinotropic effect of dairy protein have reported that the whey protein fraction contains the predominant insulinotropic secretagogue [47]. Whey protein is a rapidly digested fast dietary protein that typically induces a large initial increase in postprandial blood amino acid concentrations [38]. Individual amino acids are known to act as potent insulin secretagogues through their effects on pancreatic β cells [48,49]. Specifically, the amino acids leucine, isoleucine, valine, lysine, and threonine have been proposed as the most likely amino acids responsible for the increase in postprandial insulin concentrations [50]. In this study, the ingestion of 31.9 g WPC (containing 25 g amino acids and 1.28 g carbohydrate), and subsequent increase in postprandial plasma insulin concentrations may explain the relative maintenance of blood glucose concentration during the postprandial period. In contrast to the present findings, Paulussen et al. [22] reported that plasma glucose concentrations decreased after the ingestion of 25 g pea protein isolate (containing 20 g amino acids and 0.21 g carbohydrate), but with higher plasma glucose concentration when pea protein was co-ingested with P3 than that with placebo. It was suggested that the P3 mixture of wild-type enzyme preparations may have demonstrated side activities: specifically, amylase and glucoamylase enzymatic responses that digested carbohydrates present in the pea protein.

In this study, we also examined aspects of appetite control including changes in select gut-derived appetite regulatory hormone concentrations, VAS-derived appetite sensations, and subsequent ad libitum meal energy intake. The consumption of dietary protein may stimulate satiety more than dietary carbohydrate or fat [14,31,51]. These satiety responses following protein ingestion are typically accompanied by changes in gut-derived anorexigenic hormones, including PYY and GLP-1, and the orexigenic hormone ghrelin [51], which are released from the GI tract in response to the presence of amino acids [52]. Although the mechanism(s) underpinning the satiating effect of dietary protein are not entirely clear, it may at least partially relate to the postprandial increase in circulating amino acid concentrations [5] and/or alterations in appetite hormones [53]. In the 1950s, Mellinkoff et al. [5] reported that a postprandial increase in amino acid concentrations in the blood was accompanied by a reduction in appetite, whereas an increase in appetite was accompanied by a decline in blood amino acid concentrations. In support of this notion, Hall et al. [54] reported that whey protein ingestion resulted in a 28% increase in postprandial plasma amino acid concentration and a 19% reduction in energy intake at a subsequent test meal compared with slowly digested casein protein. Other studies incorporating alternative sources of dietary protein, leading to differences in postprandial plasma amino acid concentration, have also reported differences in gut-derived appetite regulatory hormone concentration [53,54]. Therefore, differences in postprandial plasma amino acid concentration after WPC co-ingestion with P3 may influence circulating concentrations of anorexigenic and/or orexigenic hormones, resulting in altered satiety and subsequent energy intake. In this study, we found that the time–response profile for postprandial plasma active ghrelin concentration was 12% lower in P3 than that for placebo; however, there were no treatment-dependent differences in postprandial plasma active GLP-1 or total PYY concentration (Figure 10). Ghrelin is thought to be an orexigenic hormone primarily released by the stomach that is often termed the “hunger hormone.” A recent systematic review and meta-analysis [55] reported that ghrelin is associated with perceptions of hunger in humans, and this relationship is enhanced when acylated ghrelin is examined. Alternatively, GLP-1 and PYY are anorexigenic hormones commonly measured in acute meal feeding studies and are responsive to protein ingestion [51]. Given that GLP-1 function is acutely regulated and inhibited by endogenous dipeptidyl peptidase IV activity [56], these data also provide support for the safety of P3 supplementation, in that the postprandial plasma time–response profile of GLP-1 did not differ between P3 and placebo.

In addition to treatment-dependent differences in the time–response profile of postprandial plasma active ghrelin concentration, there were treatment-dependent differences in the time–response profile of select VAS-derived appetite measures including subjective fullness and satiation (Figure 11C, E). Specifically, co-ingestion of WPC with P3 resulted in significantly greater fullness than placebo (treatment: *P* = 0.026). Furthermore, co-ingestion of WPC with P3 resulted in significantly greater satiety than placebo from 30 to 150 min in the postprandial period (all *P* < 0.05). The greater increase in subjective ratings of fullness and satiation with co-ingestion of WPC with P3 than that in placebo may partially relate to the lower postprandial plasma active ghrelin concentrations in P3. It is also plausible that the greater early (60-min) postprandial plasma EAA and BCAA iAUC with co-ingestion of WPC and P3 than that of placebo could have influenced subjective fullness and satiation, in partial agreement with the aminostatic theory by Mellinkoff et al. [5]. Finally, it is also possible that other appetite regulatory hormones, not measured in this study, such as cholecystokinin or leptin, contributed to this difference in subjective fullness and satiation.

Despite treatment differences in the time–response profile of subjective fullness and satiation in this study, there was no difference in ad libitum meal energy intake with co-ingestion of WPC with P3 than that with placebo when assessed 240-min following treatment administration. This result aligns with the lack of difference between P3 and placebo treatments at the 240-min time point on all amino acid, appetite regulatory hormone, and subjective VAS-derived appetite sensation outcomes in this study. In future studies, provision of the ad libitum test meal earlier in the experimental period (i.e., when subjective ratings of appetite are different) is warranted to test for potential differences in ad libitum energy intake.

This clinical trial has several strengths, including the rigorous randomized, double-blind, placebo-controlled, crossover design, and thorough examination of primary and secondary outcome variables. However, this clinical trial has limitations. Although our results demonstrate acute effects of protease co-ingestion with WPC on select amino acid outcomes, the effects were subtle and key metabolic responses such as changes in skeletal muscle and/or whole-body protein metabolism were not evaluated. Although there were treatment-dependent differences in perceptions of fullness and satiation based on VAS, meal energy intake was not different between treatments. By administering the ad libitum meal at 240 min, potential differences in meal energy intake between P3 and placebo treatments at earlier timepoints may have been missed. Also unclear is whether these acute plasma aminoacidemia and appetite findings are clinically meaningful regarding muscle health (e.g., muscle mass and strength), BMI modulation, and body weight change following chronic P3 supplementation. Despite these limitations, the present findings offer important insights into the role of microbial proteases and their effects on postprandial plasma amino acid concentrations, appetite, and satiety when co-ingested with whey protein, and provide a strong foundation for future studies.

In conclusion, results from this study demonstrate that acute WPC co-ingestion with P3 increased postprandial plasma EAA and BCAA 60-min iAUC compared with placebo. In terms of appetite related outcomes, the time–response profile of postprandial plasma active ghrelin concentration was lower, whereas VAS-derived sensations of fullness and satiation were greater, when WPC was co-ingested with P3 than that of placebo. Future human studies are required to determine the metabolic effects of these early (i.e., 60-min) postprandial differences in plasma EAA and BCAA iAUC, for example, on whole-body and skeletal muscle protein synthesis rates. Additional studies are also warranted in older and/or clinical populations who may have compromised protein digestion/absorption due to aging and/or medication use. Aging is associated with impaired mastication, delayed gastric emptying, and decreased pancreatic enzyme output with reduced concentrations of the major proteolytic enzymes [57]. In these populations, even a subtle improvement in the capacity to digest dietary protein and increase postprandial plasma amino acid availability could be of significance from a nutrition perspective. Finally, longer-term P3 supplementation trials within the context of a mixed meal setting, both laboratory based and free living, examining metabolic and body composition outcomes are required.

## Author contributions

The authors’ responsibilities were as follows – TAC-V, SMG, SAS, SJH, YH: contributed to the conception and design of the experiments; YH, TAC-V, SMG: contributed to drafting or revising the intellectual content of the manuscript and had primary responsibility for the final content; YH, ZWB, AA, BS, DM, TK-I, JAM, KMT: contributed to the collection of data; YH, AG, BS, SG, TAC-V, SMG: contributed to the analysis and interpretation of data; KMT, JAC: performed statistical analysis; and all authors: have read, edited, and approved the final version of the manuscript.

## Data availability

Data described in the manuscript will be made available upon request pending application and approval from the corresponding author.

## Funding

This study was funded by BIO-CAT, Inc. The funders had a role in the design of the study, in the collection and analysis of in vitro data only, in the interpretation of data, in the writing of the manuscript, and in the decision to publish the results. TAC-V was supported by the Canada Foundation for Innovation (CFI) John R. Evans Leaders Fund (Project 40372).

## Conflict of interest

This project was funded by BIO-CAT, Inc. SMG and KMT are employees of BIO-CAT, Inc, which provided the investigational products and distributes the active product under the trade name OPTIZIOME P3 HYDROLYZER. BIO-CAT, Inc, filed a patent application related to the enclosed findings (United States Patent Application No. 18/552254). SAS is an employee of Iovate Health Sciences International, Inc, which has marketed and sold MuscleTech Plant Protein formulated with OPTIZIOME P3 HYDROLYZER. BIO-CAT and Iovate stand to benefit financially from research of proteases and whey protein. SMG and SAS were involved in the design of the study, in the interpretation of data, and in the writing of the manuscript. KMT was involved in the collection of in vitro data only. TAC-V reports funding grants from BIO-CAT, Inc. The other authors report no conflicts of interest.
